# Effectiveness of transcutaneous electrical acupoint stimulation for postoperative nausea, vomiting and pain in cancer patients: a systematic review and meta-analysis of randomized controlled trials

**DOI:** 10.3389/fmed.2026.1772210

**Published:** 2026-02-13

**Authors:** Jie Chu, Jiangxue You, Ruiqi Li, Zhe Wu, Chang Liu, Chao Tian

**Affiliations:** 1 School of Medicine, University of Electronic Science and Technology of China, Chengdu, China; 2 Department of Breast Surgery, Affiliated Hospital of Southwest Medical University, Luzhou, China; 3 Frontier Medical Equipment Research Institute of Chengdu Tianfu Jincheng, Chengdu, China; 4 Department of Breast Surgery, Sichuan Clinical Research Center for Cancer, Sichuan Cancer Hospital and Institute, Sichuan Cancer Center, University of Electronic Science and Technology of China, Chengdu, China

**Keywords:** cancer, meta-analysis, PONV, postoperative pain, systematic review, transcutaneous electrical acupoint stimulation (TEAS)

## Abstract

**Objective:**

To evaluate the effectiveness of transcutaneous electrical acupoint stimulation (TEAS) in relieving postoperative pain and reducing the incidence of postoperative nausea and vomiting (PONV) in patients undergoing cancer surgery.

**Methods:**

A systematic search was conducted in PubMed, Web of Science, Embase, the Cochrane Library, CNKI, and Wanfang databases to identify randomized controlled trials (RCTs) published between January 2015 and May 2025. Postoperative pain scores at different time points, assessed using the visual analog scale (VAS) or numerical rating scale (NRS), as well as the incidence of PONV, postoperative nausea (PON), and postoperative vomiting (POV), were extracted. Subgroup analyses were performed according to the timing of TEAS intervention and pain assessment methods. The risk of bias was evaluated using the Cochrane Risk of Bias (RoB) tool, and the meta-analysis was conducted using RevMan 5.4 software.

**Results:**

A total of 16 randomized controlled trials involving 2,017 postoperative cancer patients were included (1,125 in the TEAS group and 892 in the control group). Meta-analysis of 13 studies showed that TEAS significantly reduced postoperative pain scores (SMD = −1.19, 95% CI: −1.42 to −0.95, *p* < 0.00001). Eleven studies indicated that TEAS decreased the incidence of PONV (RR = 0.47.95% CI:0.37~0.61, *P*<0.00001). Four studies were included in the meta-analysis of postoperative nausea, showing a significant reduction in incidence in the TEAS group compared to controls (RR = 0.33, 95% CI: 0.22 to 0.49, *p* < 0.00001). Another four studies showed a downward trend in postoperative vomiting but without statistical significance (RR = 0.69, 95% CI: 0.44 to 1.09, *p* = 0.11).

**Conclusion:**

TEAS appears to be an effective adjunctive intervention for alleviating postoperative pain and nausea in patients undergoing cancer surgery, showing clear clinical advantages. Further high-quality, large-scale, and multicenter studies are warranted to confirm its long-term efficacy and to promote the standardization of TEAS protocols for broader clinical application.

**Systematic review registration:**

https://www.crd.york.ac.uk/PROSPERO/view/CRD420251038890

## 1 Introduction

Surgical treatment is one of the primary modalities for cancer treatment, with common postoperative complications including pain, postoperative nausea and vomiting (PONV), sleep disturbances, and gastrointestinal dysfunction (1, 2). Among these, postoperative pain and PONV are considered the most prevalent symptoms, with incidence rates ranging from 30 to 80% (3–5). These complications not only exacerbate patients’ physical distress but also contribute to anxiety and depression, which can affect treatment adherence and postoperative recovery (6). Additionally, some patients may undergo chemotherapy or radiotherapy, whose side effects further impair the overall health condition (7).

In cancer surgery, especially in procedures involving extensive tissue resection or organ reconstruction, postoperative pain tends to be more severe. Conventional pain management strategies, including patient-controlled analgesia (PCA) pumps and opioid use, are commonly employed (8). While effective in pain relief, these strategies are often associated with notable adverse effects such as respiratory depression, nausea, vomiting, and tolerance (9). PONV, being a common postoperative complication, is typically managed with antiemetic drugs such as 5-HT₃ receptor antagonists; however, their effectiveness is inconsistent, and they may lead to side effects like headaches and constipation (10, 11). Therefore, exploring a safe, low side-effect, and patient-compliant non-pharmacological intervention is of significant clinical importance.

Transcutaneous Electrical Acupoint Stimulation (TEAS), a non-invasive treatment that applies low-frequency electrical stimulation through skin electrodes at specific acupoints, is often referred to as “needleless acupuncture.” Transcutaneous Electrical Nerve Stimulation (TENS) is also a non-invasive electrical stimulation technique, and in many studies, stimulation is similarly applied at defined acupoints. Both approaches are easy to administer and repeatable, and they have gained attention in perioperative pain management and PONV prevention (12–14). Several systematic reviews and meta-analyses have demonstrated that TEAS can significantly improve postoperative recovery outcomes across various surgical populations. These benefits include enhanced overall quality of recovery, reduced postoperative pain—particularly within the first 72 h after surgery—and decreased opioid consumption, highlighting TEAS as a promising non-pharmacological adjunctive intervention (15, 16). In addition, Liu et al. (17) and Wu et al. (18) demonstrated that TEAS is effective in reducing PONV, especially among patients receiving general anesthesia, further supporting its role in perioperative symptom management.

However, patients undergoing cancer-related surgery often experience a higher postoperative symptom burden due to tumor-related factors and prior treatments, such as chemotherapy. Despite this, evidence specifically addressing the effects of TEAS on postoperative pain and PONV in cancer patients remains limited. Therefore, the present study systematically assessed the efficacy of TEAS in patients undergoing cancer-related surgery, with postoperative nausea (PON) and postoperative vomiting (POV) analyzed as separate outcomes. Furthermore, this review incorporates randomized controlled trials published in the past decade, thereby enhancing the timeliness and completeness of the evidence and providing a more robust evidence base for the application of TEAS in postoperative rehabilitation among cancer patients.

## 2 Methods

### 2.1 Protocol and registration

This meta-analysis has been prospectively registered on the International Systematic Review platform, PROSPERO (registration number: CRD420251038890). A systematic literature review and quantitative synthesis approach were used to assess the effects of TEAS on postoperative pain and PONV in cancer patients. The literature selection, data extraction, and statistical analysis were conducted in accordance with the PRISMA (Preferred Reporting Items for Systematic Reviews and Meta-Analyses) statement and the AMSTAR (A Measurement Tool to Assess Systematic Reviews) guidelines for systematic review methodology and quality assessment (19–21).

### 2.2 Inclusion and exclusion criteria

Two researchers independently screened the literature. We included RCTs published from January 2015 to May 2025 that assessed TEAS for postoperative pain control and/or PONV prevention in surgical patients with cancer. TEAS was administered using surface electrodes at predefined acupoints, while comparators were conventional medication or placebo/sham stimulation. No limits were set on age, sex, ethnicity, or country. We excluded non-RCT designs (e.g., reviews, case reports, crossover trials, commentaries, and retrospective studies), animal studies, and studies in which outcome data required for meta-analysis were not available in extractable quantitative form (e.g., continuous outcomes without mean and SD/SE, or dichotomous outcomes without event counts, or outcomes reported only graphically without sufficient numerical information).

The primary outcome measures included postoperative pain (assessed using the Numerical Rating Scale [NRS] and Visual Analog Scale [VAS]) and the incidence of PONV, including PON and POV. Secondary outcomes included the use of postoperative analgesics and the reporting of TEAS-related adverse events.

### 2.3 Search strategy

We conducted systematic searches in six databases: PubMed, EMBASE, Web of Science, Cochrane Library, CNKI, and Wanfang, covering articles from database inception to May 5, 2025. The search strategy combined Medical Subject Headings (MeSH) and free text terms. The key search terms included ‘Transcutaneous Electrical Acupoint Stimulation,’ ‘Postoperative Pain,’ ‘Postoperative Nausea and Vomiting,’ and ‘Cancer’ in both English and Chinese, tailored to each database’s indexing format. Boolean operators “AND” and “OR” were used to combine terms, with adjustments made according to the characteristics of each database. To reduce publication bias, we also searched ClinicalTrials.gov and the Chinese Clinical Trial Registry, but no relevant studies were found. Additionally, we supplemented our search by reviewing the reference lists of included studies. An example of the detailed search strategy used for PubMed is provided in Supplementary File S1.

### 2.4 Literature screening and data extraction

Two researchers independently conducted the literature screening and data extraction according to the standards outlined in the Cochrane Handbook for Systematic Reviews of Interventions (22). Any discrepancies during the screening process were resolved by a third researcher, who made the final decision.

The literature screening process involved the following steps: First, we screened the titles and abstracts to exclude obviously irrelevant studies. Then, full-text articles were reviewed to further assess eligibility based on the inclusion and exclusion criteria. Finally, the reference lists of the included studies were manually searched to identify any additional relevant studies that may have been missed.

The data extracted from the eligible studies covered study details (e.g., authors, year of publication, country, and study setting), patient characteristics (sample size, age, sex, and type of surgery), and intervention information (TEAS acupoints, stimulation frequency and intensity, session duration, and treatment cycle). We also collected outcome data, including postoperative pain scores, the incidence of PONV, the number of nausea and vomiting episodes, analgesic use, and any TEAS-related adverse events. Risk of bias was assessed using the ROB tool. However, the number of nausea and vomiting episodes was not pooled due to the limited number of studies reporting this data. When key data were missing or unclear, we contacted the study authors to request the original information.

### 2.5 Risk of bias

The Cochrane Risk of Bias (ROB) assessment tool was used to evaluate the risk of bias in the included randomized controlled trials (RCTs), with two researchers independently assessing each study. The evaluation covered seven domains: randomization process, allocation concealment, blinding of interventions, blinding of outcome assessment, completeness of outcome data, selective reporting, and other potential biases. Each domain was rated as “low risk,” “high risk,” or “unclear” based on the information provided in the studies.

### 2.6 Statistical analysis

Statistical analyses were performed using RevMan 5.4 software, following the guidance of the Cochrane Handbook for Systematic Reviews of Interventions. For dichotomous variables, the relative risk (RR) and 95% confidence intervals (CIs) were used to assess the effect size. For continuous variables, the mean difference (MD) or standardized mean difference (SMD) and 95% CI were calculated. Different pain scoring tools, including the VAS and NRS, were both used to assess pain. Previous studies suggest a good correlation between the two (23), and the Cochrane Handbook recommends combining studies using different scales to assess the same continuous outcome using SMD (24). Subgroup analyses were performed based on postoperative time points and the type of pain scale used. A *p*-value of <0.05 was considered statistically significant (25).

Heterogeneity was assessed using the Chi-square test and the I^2^ statistic. If I^2^ > 50% or *p* < 0.1, significant heterogeneity was considered present, and a random-effects model was applied; otherwise, a fixed-effects model was used (26). Sensitivity analyses were performed by excluding studies with high risk of bias or those with significant heterogeneity. Funnel plots were used to assess publication bias for the primary outcomes.

## 3 Results

### 3.1 Screening results

**Figure 1:**
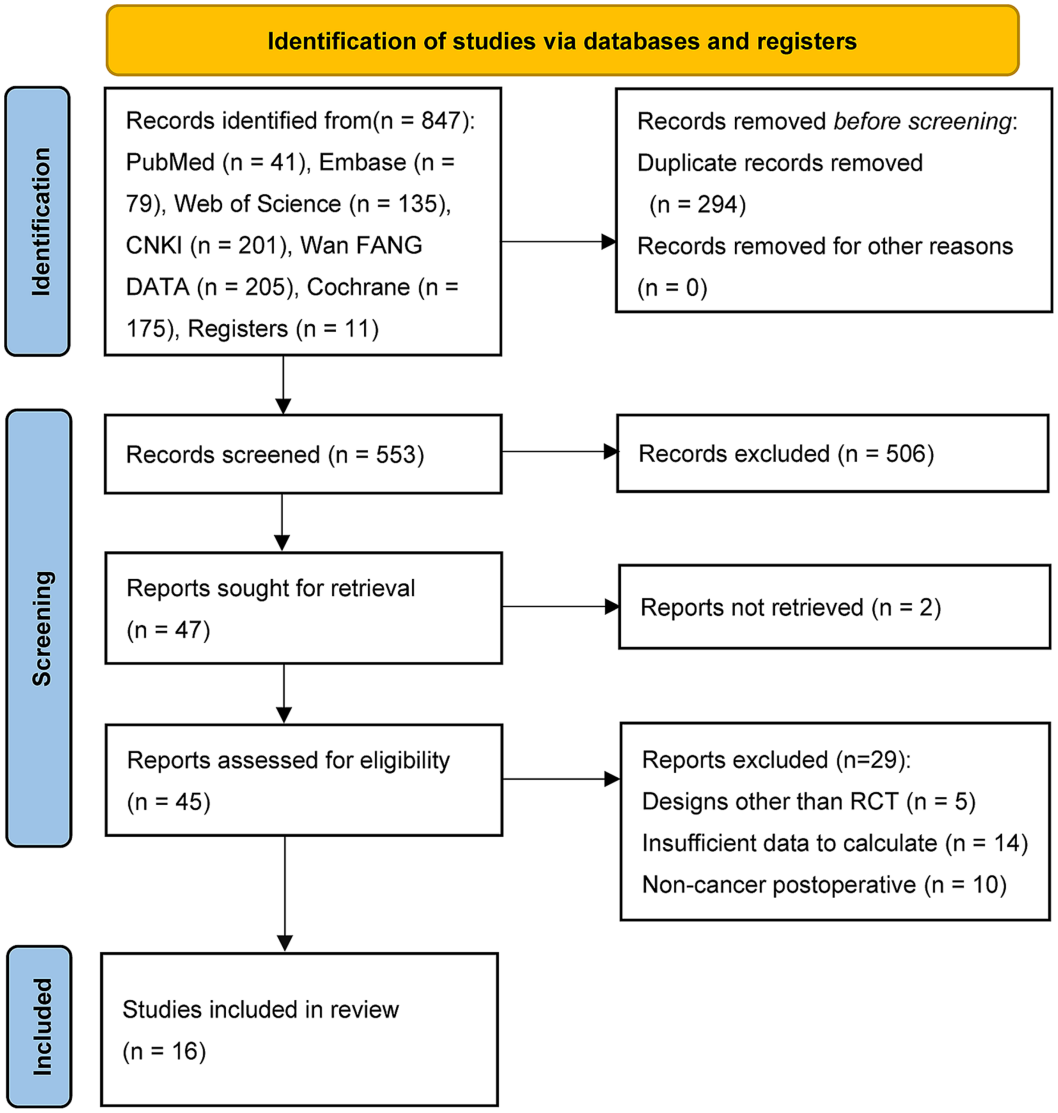
PRISMA 2020 flow diagram for new systematic reviews.

A total of 836 articles were initially identified, and after removing duplicates, 553 studies remained for title and abstract screening. After excluding studies that did not meet the inclusion criteria, 506 studies were discarded. The remaining 47 studies underwent full-text assessment, of which 2 were excluded due to unavailability of the full text. The remaining 45 studies were thoroughly evaluated against the inclusion and exclusion criteria, resulting in the inclusion of 16 RCTs for the meta-analysis (Figure 1).

### 3.2 Baseline characteristics

The 16 included RCTs involved a total of 2,017 patients, with 1,125 in the TEAS group and 892 in the control group. The average age of participants ranged from 40 to 60 years. The intervention primarily involved TEAS, with some studies combining other treatments, such as ondansetron or auricular acupressure. The control group received either conventional treatment or sham TEAS. The types of cancers included gastrointestinal tumors (6 studies), breast cancer (6 studies), lung cancer (2 studies), thyroid cancer (1 study), and cervical cancer (1 study). Geographically, 15 studies were conducted in China, while 1 study was from Turkey. The specific characteristics of the studies are summarized in Table 1.

**Table 1:** Characteristic of the included studies.

Study	Country	Surgery type	Intervention	Sample size	Mean age (years)	Outcomes*	RoB
Gu 2018 (55)	China	LRG	Control	60	58.80 ± 8.70	1,4	Unclear
			Short TEAS	60	56.90 ± 9.30		
			Long TEAS	60	60.20 ± 7.60		
Jiang 2019 (38)	China	URT	Sham TEAS + Sham auricular acupressure	31	47.00 ± 9.00	2,3,4	Unclear
			TEAS + auricular acupressure	31	44.00 ± 11.00		
Fu 2022 (36)	China	LRRC	Sham TEAS	21	59.8 ± 10.55	2,3,4	Unclear
			TEAS	25	62.0 ± 10.81		
Zhang 2020 (56)	China	ECCS	Sham TEAS	45	65.00 ± 11.00	1	Unclear
			TEAS	45	64.00 ± 12.00		
Chen 2021 (57)	China	CCS	Sham TEAS	36	47.13 ± 5.24	1,4	Unclear
			TEAS	36	45.36 ± 5.21		
Yan 2025 (28)	China	UMRM	Sham TEAS	68	48.00 ± 8.00	1,4	High
			TEAS	70	48.00 ± 8.00		
Jin 2020 (37)	China	RM	Sham TEAS	31	50.90 ± 7.10	1,4	High
			TEAS	30	48.20 ± 6.90		
Ye 2024 (58)	China	GCS	Sham TEAS	40	63.84 ± 8.50	4	Unclear
			TEAS	40	62.92 ± 8.34		
Ma 2023 (33)	China	RM	Control	60	44.79 ± 4.91	1,4	High
			TEAS	60	45.10 ± 4.96		
Liu 2019 (59)	China	MRM	control + sham teas	40	43.50 ± 5.10	1	Unclear
			ondansetron + sham teas	40	42.70 ± 7.20		
			TEAS	40	42.30 ± 6.50		
			TEAS + ondansetron	40	41.20 ± 6.40		
Lei 2024 (34)	China	VATS-RRLC	Control	33	62.95 ± 5.03	1,4	High
			TEAS	32	61.46 ± 2.47		
Li 2023 (35)	China	RHCC	Control	30	55.40 ± 10.20	1,4	High
			TAP	30	54.80 ± 9.30		
			TEAS+TAP	30	53.90 ± 9.60		
Gu 2019 (29)	China	LRG	Sham TEAS	59	56.67 ± 6.23	1,4	Low
			TEAS	58	57.59 ± 7.32		
Erden S 2022 (30)	Turkey	MRM	Control	40	57.10 ± 10.88	2,4	High
			TEAS	40	56.90 ± 10.20		
Chen 2020 (31)	China	VATS-RRLC	Sham TEAS	40	55.80 ± 3.20	2,3,4	Low
			TEAS	40	56.00 ± 3.70		
Lu 2021 (32)	China	RM	Sham TEAS	188	48.20 ± 8.20	1,2,3,4	High
			single acupoint	198	48.00 ± 9.10		
			combined acupoints	190	48.20 ± 9.00		

TEAS, transcutaneous electrical acupoint stimulation; TAP, Transversus Abdominis Plane block; LRG, Laparoscopic radical gastrectomy; URT, Unilateral Radical thyroidectomy; LRRCC, Laparoscopic radical resection for colorectal cancer; ECCS, Elective surgery for colorectal cancer; CCS, Colorectal cancer surgery; UMRM, Unilateral modified radical mastectomy for breast cancer; RM, Radical mastectomy; GCS, Gastric cancer surgery; MRM, Modified radical mastectomy for breast cancer; VATS-RRLC, Video-assisted thoracoscopic surgery radical resection for lung cancer; RHCC, Radical hysterectomy for cervical cancer; NR, not reported; *1: nausea and vomiting; 2: postoperative nausea; 3: postoperative vomiting; 4: Postoperative pain.

Table 2 summarizes the key characteristics of the TEAS interventions in the included studies, including the selected acupoints, stimulation duration, frequency, and current intensity. Commonly used acupoints included Neiguan (PC6), Zusanli (ST36), Hegu (LI4), and Sanyinjiao (SP6). Most studies initiated TEAS intervention 30 min before surgery, with some continuing during or after surgery. The electrical stimulation frequency was mostly in the form of sparse-dense waves, with the current intensity typically ranging from 2 to 30 mA. Most studies did not report any TEAS-related adverse events, though a few studies mentioned mild discomfort at the electrode site.

**Table 2:** TEAS stimulation parameters.

Study	Acupoints	Time point	Frequency	Adverse events	Device	Post-operative medication
Gu 2018 (55)	PC6, ST36	short TEAS-throughout the procedure; longTEAS-1 h before the procedure to 30 min postoperatively;2d, 30 min/session, 3 times/d	2/100 Hz; 8~12 mA; 0.2~0.6 ms	NR	HANS-LH402 (Beijing Pukang Pharmaceutical Technology Development Co. Ltd.)	PCIA
Jiang 2019 (38)	LI4, PC6	30 min before the procedure; throughout the procedure	2/100 Hz; 6~12 mA	NR	HANS-200A (Batch No. 200110514089)	NR
Fu 2022 (36)	ST36, SP6	30 min before the procedure	2/100 Hz; maximum tolerable level minus 1 mA	NR	HANS-200A (Nanjing Jisheng Medical Technology Co. Ltd. Nanjing, China)	PCIA
Zhang 2020 (56)	ST36, SP6, ST37	30 min after the procedure;3d, 30 min/session, 1 times/d	2 Hz; 10~25 mA	NR	TEAS device (not specified)	NR
Chen 2021 (57)	PC6, ST36	30 min before surgery to 30 min after surgery;2d, 30 min/session; 3times/d	2/100 Hz; 8~12 mA; 0.2 ~ 0.6 ms	NR	TEAS device (not specified)	NR
Yan 2025 (28)	PC6, ST36, CV17	30 min before the procedure;1d, 30 min/session, 1 times/d	2/100 Hz; Maximally tolerated intensity	NR	SDZ-V electroacupuncture device	PCIA
Jin 2020 (37)	LI4, PC6, ST36, SP6	30 min before the procedure, throughout the procedure	2/100 Hz; 6~9 mA	NR	HANS-200A (Nanjing Jisheng Medical Technology Co. Ltd. Nanjing, China)	PCIA
Ye 2024 (58)	LI4, PC6, ST36	1d before the procedure;7d, 30 min/session, every 12 h	2/100 Hz; 8~12 mA; 0.2~0.6 ms	NR	KD-2B (Beijing Yaoyang Kangda Medical Instrument Co. Ltd. Beijing, China)	NR
Ma 2023 (33)	LI4, PC6, ST36	30 min before the procedure	2/30 Hz; 6~10 mA	NR	Huatuo SDZ-V electroacupuncture device	PCIA
Liu 2019 (59)	ST36, PC6	30 min before the procedure	2/10 Hz; Maximally tolerated intensity	NR	Huatuo SDZ-V (Suzhou Medical Appliance Factory Co. Ltd. Suzhou, China)	PCIA
Lei 2024 (34)	LI4, PC6, ST36	throughout the procedure	2/100 Hz; 30 mA	NR	TEAS device(not specified)	PCIA
Li 2023 (35)	ST36, SP6	After the procedure	2/10 Hz; Maximally tolerated intensity	NR	SDZ-V (Suzhou Medical Appliance Factory Co. Ltd. Suzhou, China)	PCIA
Gu 2019 (29)	ST36, PC6	30 min before the procedure to 30 min after surgery; postoperative 2d, 30 min/session, 3 times/d	2/100 Hz; 5~30 mA	NR	HANS LH-202 Transcutaneous Electrical Acupoint Stimulation device	Analgesics (not specified)
Erden S 2022 (30)	NR	Twice postoperatively(20 min/session)	85 Hz; 50~250 ms	NR	Dual-channel TENS device (Intelect)	NR
Chen 2020 (31)	LI4, PC6, SI3, TE6	30 min before the procedure; throughout the procedure; 6, 24 and 48 h after the procedure(30 min/session)	2/100 Hz; 10–15 mA; 30 mA	NR	HANS-200A (Nanjing Jisheng Medical Technology Co. Ltd. Jiangsu, China)	sufentanil
Lu 2021 (32)	PC6, CV17	30 min before the procedure	2/10 Hz	discomfort in the skin area attached to the electrodes	Hwato (Model SDZ-V, Suzhou Medical Appliance Factory, Suzhou, China)	Sufentanil, Parecoxib sodium 40 mg

Hz, hertz; h, hour; d, day; mA, milliampere; min, minute; ms, millisecond; NR, not reported; TEAS, transcutaneous electrical acupoint stimulation; PCIA, Patient-Controlled Intravenous Analgesia; PC6, Neiguan; CV17, Danzhong; LI4, Hegu; SI3, Houxi; SP6, Sanyinjiao; TE6, Zhigou; ST37, Shangjuxu; ST36 Zusa.

### 3.3 Risk of bias

**Figure 2:**
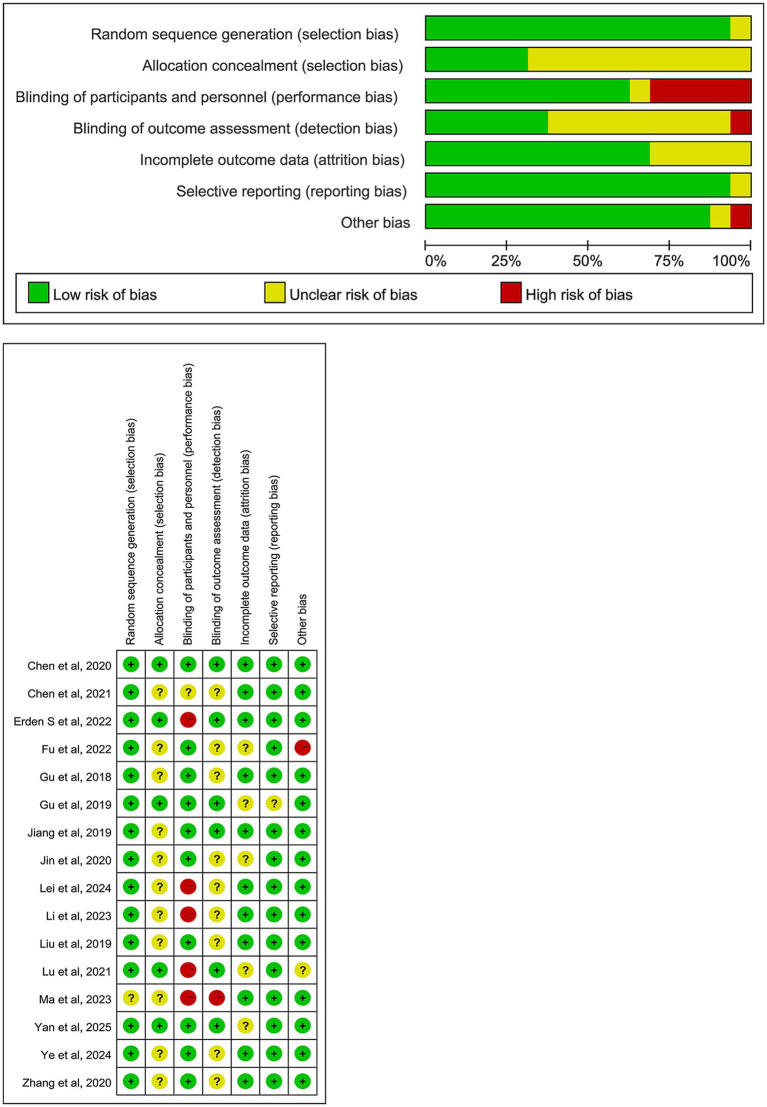
Risk of bias summary.

In this study, the Cochrane Risk of Bias assessment tool was used to evaluate the risk of bias in the included RCTs (27). The results are shown in Figure 2. Most studies were classified as low risk for randomization process, completeness of outcome data, selective reporting, and other potential biases. However, there were differences in the risk of bias across some domains in the studies.

Regarding allocation concealment, only 5 RCTs explicitly reported the allocation concealment method and were rated as low risk (28–32), while the remaining studies did not report specific methods and were classified as “unclear risk.” In terms of performance bias, about 20 to 30% of studies were rated as high risk or unclear risk (30, 32–35). Given the nature of TEAS and other physical interventions, blinding of both participants and researchers was challenging in these trials, leading to potential bias.

For outcome assessment blinding, 6 RCTs reported blinding procedures, with only one study not specifying whether blinding was implemented (33). Concerning the completeness of outcome data, 5 studies had missing data or attrition, and were rated as unclear risk (28, 29, 32, 36, 37), while the remaining studies had complete data. The vast majority of studies were rated as low risk for selective reporting and other potential biases. Overall, 1 study was classified as low risk across all domains (31), 3 studies had only 1 domain with unclear or high risk (28, 30, 38), and the remaining studies were of acceptable overall quality.

**Figure 3:**
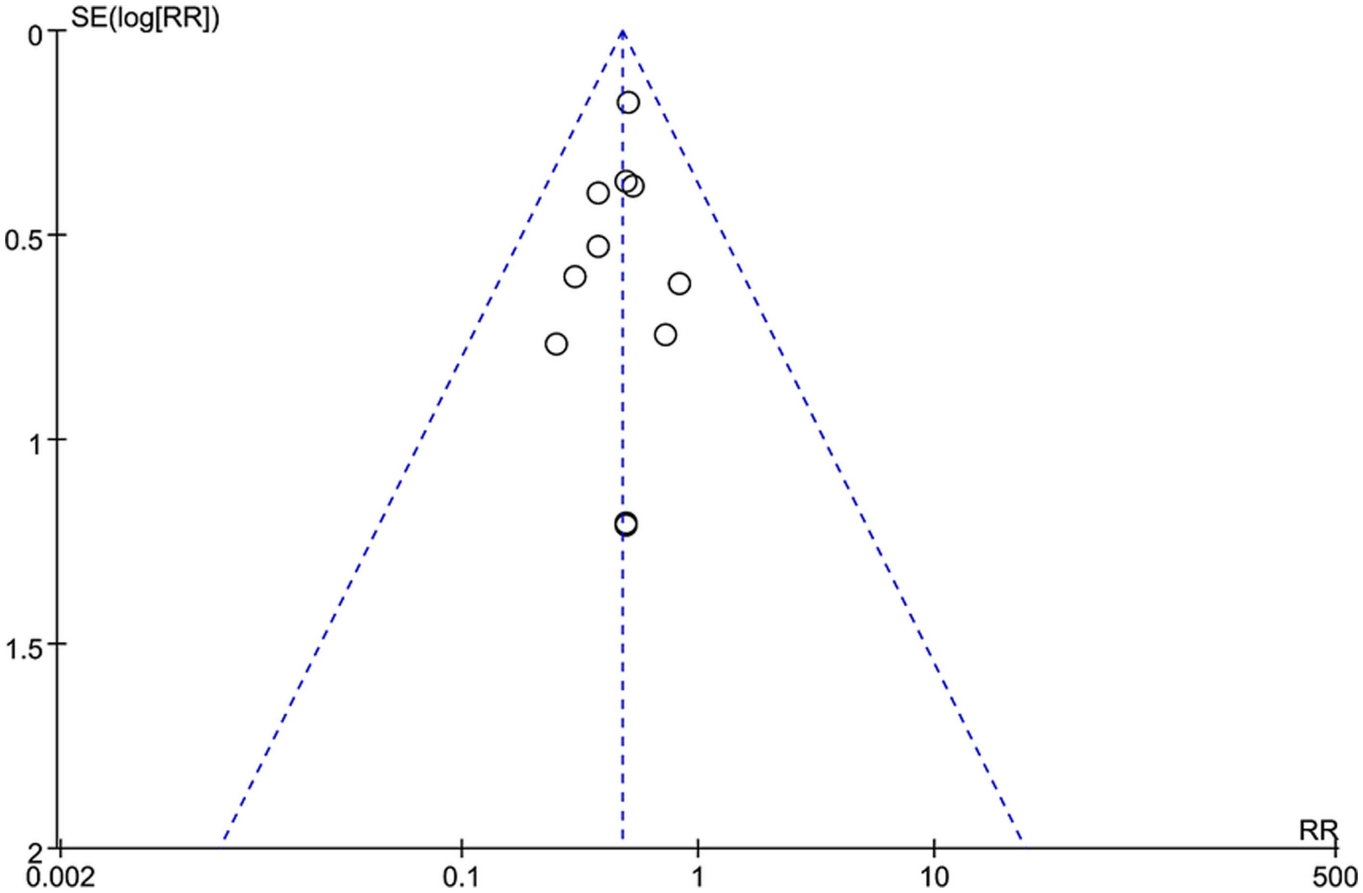
Funnel plot of PONV incidence.

Sensitivity analyses were performed by excluding studies with high or unclear risk of bias to assess their impact on the overall results. The analysis showed that excluding individual studies did not change the direction of the combined effect, indicating the robustness of the results. Publication bias was assessed using funnel plots. As shown in Figure 3, the funnel plot distribution was nearly symmetrical, suggesting a low likelihood of significant publication bias.

### 3.4 Meta-analysis results

#### 3.4.1 Postoperative pain

A total of 14 studies were included in the meta-analysis of postoperative pain, comprising 742 patients in the TEAS group and 737 in the control group. There was significant heterogeneity among the included studies (I^2^ = 88%, *p* < 0.00001), and therefore a random-effects model was used. The pooled results indicated that patients in the TEAS group experienced significantly reduced postoperative pain compared to those in the control group (SMD = −1.19, 95% CI [−1.42, −0.95], *p* < 0.00001).

Subgroup analyses based on different postoperative time points and pain assessment tools (VAS and NRS) were performed. The VAS results demonstrated that TEAS significantly reduced pain at 4 h (SMD = −1.11, 95% CI [−1.58, −0.64], *p* < 0.00001), 8 h (SMD = −1.04, 95% CI [−1.24, −0.84], *p* < 0.00001), 24 h (SMD = −2.18, 95% CI [−2.98, −1.37], *p* < 0.00001), and 48 h (SMD = −1.28, 95% CI [−1.84, −0.71], *p* < 0.00001) after surgery.

**Figure 4:**
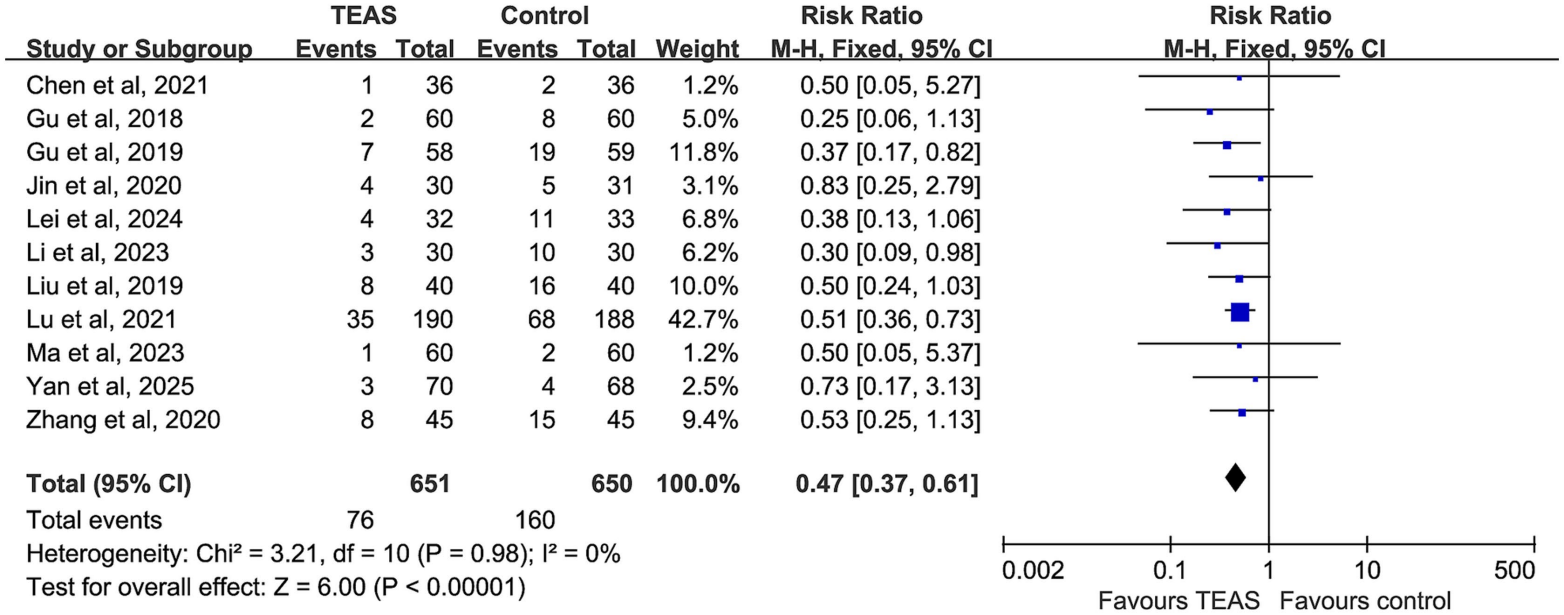
Forest plot comparing the incidence of postoperative nausea and vomiting (PONV) between TEAS and control group.

Regarding the NRS scores, TEAS was also effective in reducing pain at 6 h (SMD = −1.00, 95% CI [−1.38, −0.61], p < 0.00001), 12 h (SMD = −0.85, 95% CI [−1.23, −0.47], *p* < 0.00001), and 24 h (SMD = −0.60, 95% CI [−1.09, −0.11], *p* = 0.02), postoperatively (Figure 4).

#### 3.4.2 Incidence of postoperative nausea and vomiting

**Figure 5:**
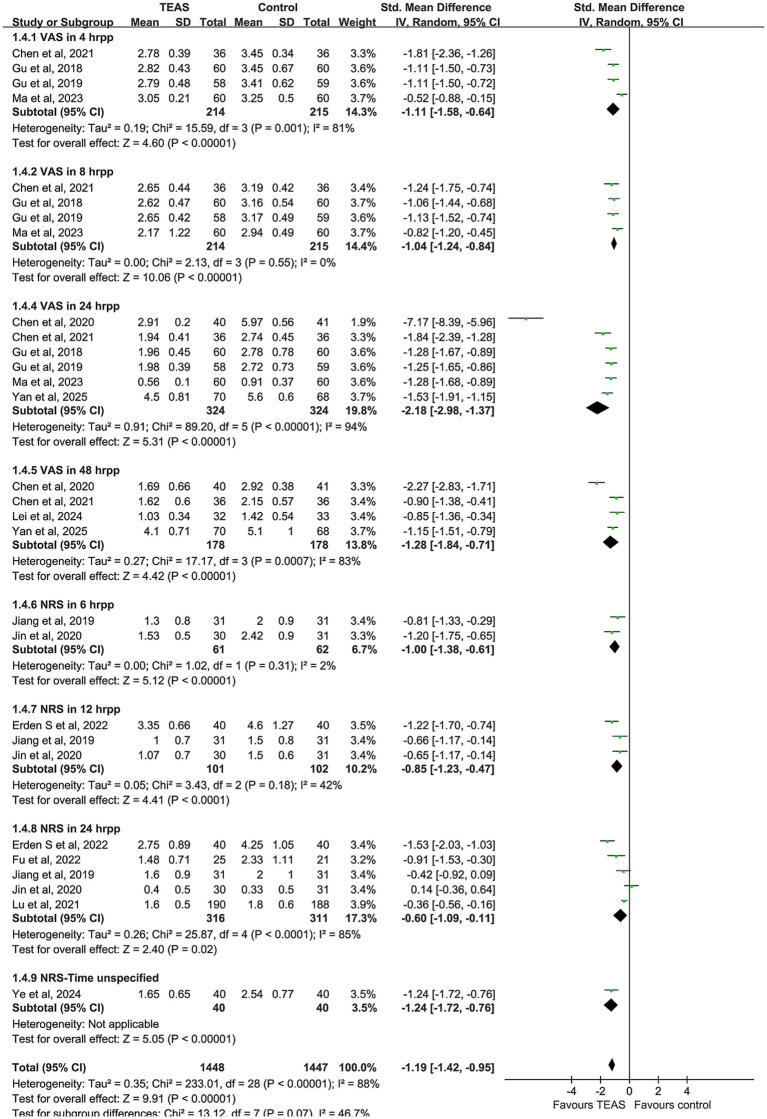
Forest plot comparing the impact of TEAS group and control group on postoperative pain based on postoperative time and measurement tools.

A total of 11 studies reported the incidence of PONV, involving 651 participants in the TEAS group and 650 in the control group. No evidence of heterogeneity was observed among studies (*p* = 0.98, I^2^ = 0%), and a fixed-effect model was applied. The pooled analysis revealed that TEAS significantly reduced the incidence of PONV compared with the control group (RR = 0.47, 95% CI: 0.37–0.61, *p* < 0.00001) (Figure 5).

#### 3.4.3 Incidence of postoperative nausea

**Figure 6:**
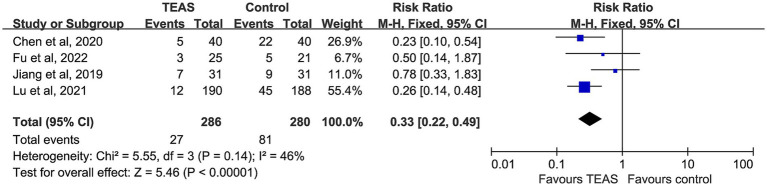
Forest plot comparing the incidence of postoperative nausea (PON) between TEAS and control group.

Five studies reported the incidence of postoperative nausea. However, the study by Sevilay ERDEN (2022) assessed nausea using a scoring scale rather than actual event counts, and thus was excluded from quantitative synthesis. Ultimately, data from 286 patients in the TEAS group and 280 in the control group were analyzed. Moderate heterogeneity was present but not statistically significant (I^2^ = 46%, *p* = 0.14), and a fixed-effect model was used. The results indicated a significantly lower incidence of postoperative nausea in the TEAS group compared with the control group (RR = 0.33, 95% CI: 0.22–0.49, *p* < 0.00001) (Figure 6).

#### 3.4.4 Incidence of postoperative vomiting

**Figure 7:**
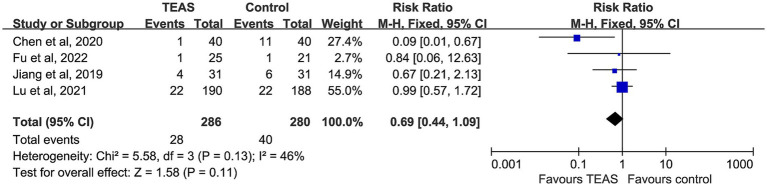
Forest plot comparing the incidence of postoperative vomiting (POV) between TEAS and control group.

Four studies compared the incidence of postoperative vomiting, including 286 participants in the TEAS group and 280 in the control group. No significant heterogeneity was detected (I^2^ = 46%, *p* = 0.13), and a fixed-effect model was adopted. Although the TEAS group had a relatively lower incidence of vomiting, the difference was not statistically significant (RR = 0.69, 95% CI: 0.44–1.09, *p* = 0.11) (Figure 7).

## 4 Discussion

This study included 16 RCTs involving cancer patients post-surgery. Significant heterogeneity was observed only in the postoperative pain outcome, and no apparent publication bias was detected. The meta-analysis demonstrates that TEAS significantly alleviates postoperative pain and reduces the incidence of PONV, showing high clinical potential. It can help reduce patient discomfort and lower the risk of postoperative complications.

Currently, postoperative pain and PONV in cancer patients are primarily managed with pharmacological treatments. Opioids such as morphine and fentanyl are commonly used for pain relief. While these drugs provide rapid pain relief, they are associated with adverse effects such as respiratory depression, tolerance, nausea, vomiting, and excessive sedation (9). For PONV, 5-HT₃ receptor antagonists like ondansetron are frequently used, but these medications can cause side effects such as headaches, constipation, and QT prolongation (10). Some cancer patients may have poor responses to pharmacological treatments or may have contraindications, which further complicates postoperative management. Compared to general surgical patients, cancer patients often have more extensive surgical trauma, as well as comorbidities such as malnutrition, anemia, and impaired liver and kidney function, leading to poor opioid tolerance and a higher risk of adverse reactions like constipation and respiratory depression (39, 40). Additionally, some patients may have a history of perioperative chemotherapy, which significantly increases the risk of recurrent PONV post-surgery (41), further highlighting the importance of non-pharmacological interventions.

In this context, TEAS, a non-pharmacological treatment combining traditional Chinese medicine meridian theory and modern electrical stimulation techniques, has gained attention for its non-invasive, cost-effective, easy-to-operate, and low-equipment and environmental requirements. It has gradually been applied in perioperative management (42). Administered by trained physicians or nurses at different stages (preoperative, intraoperative, or postoperative), TEAS helps alleviate postoperative pain and PONV without adding to the pharmacological burden. It effectively reduces opioid and antiemetic medication usage and related side effects, making it particularly valuable for cancer patients, a high-risk group. TEAS has the potential to become an essential component of postoperative pain and PONV management for cancer patients. Future studies could include outcomes related to medication dosage and opioid-related adverse events to provide a more comprehensive evaluation of TEAS in ERAS (Enhanced Recovery After Surgery) protocols for cancer patients during the perioperative period.

Previous systematic reviews on acupoint stimulation for PONV prevention have mostly focused on mixed surgical or anesthetic populations (43, 44). These studies mainly assess the overall incidence of PONV but lack a systematic exploration of the differences in PON and POV in cancer patients. In contrast, this study focused on cancer surgical patients and separately evaluated the effects of TEAS on postoperative pain, PON, and POV. These findings contribute to expanding the evidence base for the application of TEAS in perioperative oncology care.

### 4.1 Pain

This study demonstrates that TEAS significantly alleviates postoperative pain, which is consistent with previous research. Subgroup analysis indicated that VAS and NRS scores at different time points (4 h, 24 h, and 48 h postoperative) were significantly lower in the TEAS group compared to the control group, with the most significant effect observed at 24 h post-surgery. The effective pain relief not only helps reduce discomfort but also facilitates early mobilization and functional recovery of the patients.

Regarding acupoint selection, LI4 (Hegu) and ST36 (Zusanli) were the most commonly used acupoints for pain relief. Research has shown that stimulation of LI4 and ST36 can regulate pain transmission and perception by activating the hypothalamus-pituitary–adrenal axis and enhancing the release of endogenous opioid peptides. It also reduces pain sensitivity by downregulating TRPV1 and associated neuroinflammatory signals (45–47). Most studies used an alternating frequency of 2/100 Hz, combining the dual mechanisms of low-frequency stimulation to induce the release of encephalins and high-frequency stimulation to inhibit the release of substance P, which helps to enhance the analgesic effect (48). Regarding the timing of intervention, a preoperative start 30 min before surgery with continuation into the postoperative period was associated with better analgesic effects. This suggests that continuous electrical stimulation may help reduce the postoperative pain peak and opioid consumption, indicating that the timing of intervention could be a key factor influencing efficacy (49).

Postoperative pain in cancer patients is often accompanied by chronic inflammation and opioid tolerance. TEAS offers an advantage by reducing pain and decreasing opioid use through a non-pharmacological approach. This study exhibited high heterogeneity in postoperative pain outcomes, which is common in meta-analyses of pain-related outcomes. The sources of heterogeneity may include variations in TEAS stimulation parameters, acupoint selection, the use of adjunctive analgesic medications, different postoperative assessment time points, and the types of surgeries performed. However, the heterogeneity did not significantly affect the direction of the effect, as all studies indicated that TEAS had a positive impact on postoperative pain. Therefore, while this conclusion is meaningful, the effect size should be interpreted with caution in light of the observed heterogeneity.

### 4.2 Nausea and vomiting

The results of this study show that TEAS significantly reduces the incidence of PONV and PON in cancer patients, with low heterogeneity between studies. However, while there was a trend towards a reduction in POV, the difference did not reach statistical significance. This may be due to the different neuroregulatory pathways involved in PON and POV. Nausea primarily involves the cortical–limbic system and is a subjective experience. TEAS can suppress excessive gastrointestinal motility, regulate serum 5-HT levels, and modulate vagal nerve activity, which reduces nausea (50), while maintaining higher postoperative serum ghrelin levels to enhance gastrointestinal motility, thus reducing the incidence of PONV (51). In contrast, vomiting is a complex somatic reflex behavior coordinated by the brainstem and the vomiting center (52). The modulation of this reflex pathway by TEAS may not be as sensitive as its effect on nausea, resulting in limited efficacy. Controlling vomiting may require interventions that directly target the brainstem reflex mechanism, which warrants further investigation in future studies.

Differences in acupoint selection across studies may also influence POV outcomes. For example, Chen et al. successfully reduced postoperative vomiting incidence in lung cancer patients by stimulating LI4, PC6, SI3, and TE6, while Lu et al. using a combination of PC6 and CV17 did not observe significant improvements. This suggests that the choice of acupoints may play a role in the therapeutic outcomes. Additionally, the small sample sizes in some studies may limit the statistical power. Overall, TEAS was found to have more consistent efficacy in alleviating nausea.

The frequency of stimulation in the studies we included was primarily 2/100 Hz, but Liu et al. used a low-frequency 2/10 Hz. Both frequencies demonstrated some efficacy, though there is no established standard for frequency at this time. Notably, in studies involving laparoscopic surgery, TEAS significantly alleviated PONV, which may be related to the specific injury pattern and anesthesia management associated with this type of surgery. This suggests that future research should explore the impact of surgical techniques on TEAS efficacy.

While different cancer types may influence the baseline risk of PONV, no significant differences were found in the subgroup analysis, indicating that TEAS may offer consistent efficacy across different cancer types post-surgery. However, it should be noted that some subgroups had small sample sizes, and the results require further validation.

Safety and tolerability are important considerations for the clinical application of TEAS. Previous studies have shown that TEAS-related adverse events are generally limited to mild local skin reactions, such as irritation, redness, tingling, or itching, which may be more likely to occur in patients with sensitive skin or during prolonged use. These discomforts can be effectively minimized through appropriate selection of electrode materials, standardized electrode placement, adjustment of stimulation intensity, and regular assessment of skin condition, thereby improving patient adherence. Across the randomized controlled trials included in the present review, TEAS-related adverse events were infrequently reported. Only one patient in the study by Lu et al. (32) experienced mild local discomfort at the stimulation site, including itching and erythema, which resolved spontaneously without treatment interruption. Overall, current evidence indicates that TEAS-related discomfort is typically mild, transient, and self-limiting. Its favorable safety profile and non-invasive nature support TEAS as a feasible adjunctive option for postoperative symptom management, particularly in cancer patients with a high symptom burden.

Several limitations of the current study must be acknowledged. First, most of the included studies had small sample sizes, which may reduce the stability of the analysis. Second, there was significant heterogeneity in the postoperative pain outcomes, possibly due to differences in intervention parameters, assessment tools, and cancer types. Furthermore, 15 of the included studies were from China, which introduces regional limitations. This means that our findings primarily reflect the unique medical environment, clinical practices, and healthcare culture specific to China. The influence of traditional Chinese medicine, the proficiency of healthcare providers, and patients’ acceptance of treatments may differ substantially from those in other countries. Additionally, variations in surgical techniques and postoperative care protocols across countries could influence the effectiveness of TEAS in different regions. As a result, the generalizability of our conclusions is limited, and they are most applicable to regions with similar medical and cultural contexts. In addition, another important limitation is the lack of consideration for the combined use of TEAS with other traditional Chinese medicine (TCM) therapies in clinical practice. In actual clinical settings, TEAS is often used alongside TCM treatments such as topical herbal applications, herbal formulas, or acupoint compresses to enhance effectiveness. However, the studies included in this meta-analysis did not report data on such combinations, which may represent a potential confounding factor. Different herbal formulas and their interactions with TEAS could vary by region. Although literature suggests that TCM may enhance the effects of TEAS, this aspect remains underexplored (53, 54). Future research should investigate the combined effects of TEAS and TCM treatments, with multi-center, standardized research designs to assess their synergistic effects. This will provide a stronger evidence base for the broader application of TEAS in clinical practice. Although the study systematically searched major Chinese and English-language databases and supplemented searches in ClinicalTrials.gov and ChiCTR, no grey literature meeting the inclusion criteria was found, meaning some unpublished or ongoing studies may have been missed. Therefore, future studies should prioritize multicenter, large-scale randomized controlled trials with standardized TEAS protocols and outcome measures, conducted across diverse geographic regions and healthcare systems to improve generalizability. In addition, the potential synergistic effects of TEAS combined with traditional Chinese medicine therapies warrant further investigation using standardized study designs.

Nevertheless, the meta-analysis in this study provides strong support for the use of TEAS in postoperative pain and PONV management in cancer patients. The effectiveness of TEAS in reducing postoperative pain and overall PONV, particularly nausea, has been confirmed, and it demonstrates good safety and tolerability. Given the significant impact of pain and PONV on postoperative recovery and quality of life in cancer patients, TEAS, as a safe, non-invasive, and patient-compliant intervention, is particularly suitable for this population and holds promising potential for broader application. Future studies should further explore its efficacy across different cancer types and surgical procedures, optimize acupoint combinations and stimulation parameters, and provide a basis for the development of standardized treatment protocols.

## 5 Conclusion

In summary, TEAS is a safe, non-invasive, and easy-to-administer intervention that shows promising efficacy in postoperative pain and PONV management for cancer patients. The meta-analysis results indicate that TEAS significantly reduces pain scores at multiple postoperative time points and decreases the overall incidence of PONV, as well as postoperative nausea. These effects may reduce opioid analgesic use, thus improving postoperative recovery quality.

Although this study has limitations, including regional concentration of research and inconsistent intervention protocols, the overall evidence supports the use of TEAS as a beneficial adjunctive strategy for postoperative pain and PONV management in cancer patients. Future multicenter, large-scale, rigorously designed clinical trials are needed to further validate its impact on postoperative recovery, long-term survival, and quality of life. Additionally, efforts should focus on standardizing TEAS intervention parameters and protocols to better serve clinical practice.

## Data Availability

The data analyzed in this study is subject to the following licenses/restrictions: the dataset used in this meta-analysis was extracted from published studies. No individual participant data were collected. The extracted study-level dataset and analysis files are not publicly available at this stage, but can be provided by the corresponding author upon reasonable request for academic purposes. Requests to access these datasets should be directed to CT, tc2023tc@163.com.
