# Supplementary material for: Effectiveness of transcutaneous electrical acupoint stimulation for postoperative nausea, vomiting and pain in cancer patients: a systematic review and meta-analysis of randomized controlled trials
Source: Front Med (Lausanne). 2026 Feb 13;13:1772210. doi: 10.3389/fmed.2026.1772210 (PMC12946081; doi:10.3389/fmed.2026.1772210)
Supplement: Supplementary file 1 [file Table_1.DOCX]

**Supplementary File S1.**

**Search strategy in PubMed**

The PubMed database was searched using a combination of Medical Subject Headings (MeSH) terms and free-text keywords. The following search strategy was applied:

**#1 Postoperative nausea and vomiting (PONV):** (((((((((((((("Postoperative Nausea and Vomiting"[Mesh]) OR (postoperative nausea and vomiting[Title/Abstract])) OR (nausea and Vomiting, Postoperative[Title/Abstract])) OR (PONV[Title/Abstract])) OR (Postoperative Nausea[Title/Abstract])) OR (Nausea, Postoperative[Title/Abstract])) OR (Vomiting, Postoperative[Title/Abstract])) OR (Emesis, Postoperative[Title/Abstract])) OR (Emeses, Postoperative[Title/Abstract])) OR (Postoperative Emeses[Title/Abstract])) OR (Postoperative Emesis[Title/Abstract])) OR (Postoperative Vomiting[Title/Abstract]))

**#2 Postoperative pain:** ((((((((((((((((((((((((((((((((((((((("Pain, Postoperative"[Mesh]) OR (Pain, Postoperative[Title/Abstract])) OR (Postsurgical Pain[Title/Abstract])) OR (Post-surgical Pain[Title/Abstract])) OR (Pain, Post-surgical[Title/Abstract])) OR (Post surgical Pain[Title/Abstract])) OR (Post-operative Pain[Title/Abstract])) OR (Post operative Pain[Title/Abstract])) OR (Post-operative Pains[Title/Abstract])) OR (Postoperative Pain[Title/Abstract])) OR (Pain, Post-operative[Title/Abstract])) OR (Pain, Post operative[Title/Abstract])) OR (Postoperative Pain, Acute[Title/Abstract])) OR (Pain, Acute Postoperative[Title/Abstract])) OR (Post-operative Pain, Acute[Title/Abstract])) OR (Pain, Acute Post-operative[Title/Abstract])) OR (Post operative Pain, Acute[Title/Abstract])) OR (Acute Postoperative Pain[Title/Abstract])) OR (Acute Post-operative Pain[Title/Abstract])) OR (Acute Post operative Pain[Title/Abstract])) OR (Postoperative Pain, Chronic[Title/Abstract])) OR (Pain, Chronic Postoperative[Title/Abstract])) OR (Chronic Postsurgical Pain[Title/Abstract])) OR (Chronic Postsurgical Pains[Title/Abstract])) OR (Pain, Chronic Postsurgical[Title/Abstract])) OR (Postsurgical Pain, Chronic[Title/Abstract])) OR (Persistent Postsurgical Pain[Title/Abstract])) OR (Pain, Persistent Postsurgical[Title/Abstract])) OR (Postsurgical Pain, Persistent[Title/Abstract])) OR (Chronic Post-operative Pain[Title/Abstract])) OR (Chronic Post operative Pain[Title/Abstract])) OR (Post-operative Pain, Chronic[Title/Abstract])) OR (Pain, Chronic Post-operative[Title/Abstract])) OR (Post operative Pain, Chronic[Title/Abstract])) OR (Chronic Postoperative Pain[Title/Abstract])) OR (Chronic Post-surgical Pain[Title/Abstract])) OR (Chronic Post surgical Pain[Title/Abstract])) OR (Pain, Chronic Post-surgical[Title/Abstract])) OR (Post-surgical Pain, Chronic[Title/Abstract])))

**#3 Intervention:** (((((((((((((((((((((((((((((((((("Transcutaneous Electric Nerve Stimulation"[Mesh]) OR (Transcutaneous Electric Nerve Stimulation[Title/Abstract])) OR (TEAS[Title/Abstract])) OR (Acupoint stimulation[Title/Abstract])) OR (Transcutaneous Electrical Acupoint Stimulation[Title/Abstract])) OR (Transcutaneous Nerve Stimulation[Title/Abstract])) OR (Nerve Stimulation, Transcutaneous[Title/Abstract])) OR (Stimulation, Transcutaneous Nerve[Title/Abstract])) OR (Electric Stimulation, Transcutaneous[Title/Abstract])) OR (Stimulation, Transcutaneous Electric[Title/Abstract])) OR (Transcutaneous Electric Stimulation[Title/Abstract])) OR (Percutaneous Electric Nerve Stimulation[Title/Abstract])) OR (TENS[Title/Abstract])) OR (Transdermal Electrostimulation[Title/Abstract])) OR (Electrostimulation, Transdermal[Title/Abstract])) OR (Percutaneous Electrical Nerve Stimulation[Title/Abstract])) OR (Transcutaneous Electrical Nerve Stimulation[Title/Abstract])) OR (Electrical Stimulation, Transcutaneous[Title/Abstract])) OR (Transcutaneous Electrical Stimulation[Title/Abstract])) OR (Analgesic Cutaneous Electrostimulation[Title/Abstract])) OR (Cutaneous Electrostimulation, Analgesic[Title/Abstract])) OR (Electrostimulation, Analgesic Cutaneous[Title/Abstract])) OR (Electroanalgesia[Title/Abstract])) OR (Electroanalgesias[Title/Abstract])) OR (Percutaneous Neuromodulation Therapy[Title/Abstract])) OR (Neuromodulation Therapy, Percutaneous[Title/Abstract])) OR (Percutaneous Neuromodulation Therapies[Title/Abstract])) OR (Therapy, Percutaneous Neuromodulation[Title/Abstract])) OR (Percutaneous Electrical Neuromodulation[Title/Abstract])) OR (Electrical Neuromodulation, Percutaneous[Title/Abstract])) OR (Electrical Neuromodulations, Percutaneous[Title/Abstract])) OR (Neuromodulation, Percutaneous Electrical[Title/Abstract])) OR (Neuromodulations, Percutaneous Electrical[Title/Abstract])) OR (Percutaneous Electrical Neuromodulations[Title/Abstract])))

**#4 Population:** (((("Neoplasms"[Mesh]) OR (carcinoma[Title/Abstract])) OR (cancer[Title/Abstract])) OR (tumor[Title/Abstract]))

**#5** #1 OR #2

**#6** #5 AND #3 AND #4

**Date range:** from 2015/1/1 - 2025/2/1

**Filters:** Humans; No language restrictions
